# Digital Health Technologies for Screening and Identifying Unmet Social Needs: Scoping Review

**DOI:** 10.2196/78793

**Published:** 2025-11-11

**Authors:** Emre Sezgin, Daniel I Jackson, Samantha Boch, Mattina Davenport, Micah Skeens, Millie Dolce, Bianca Frankin, Lisa K Militello, Elizabeth Lyman, Kelly Kelleher

**Affiliations:** 1 The Abigail Wexner Research Institute, Nationwide Children's Hospital, 700 Children's Dr, Columbus, OH, 43205, United States, 1 6147223179; 2 College of Nursing, University of Cincinnati, Cincinnati, OH, United States; 3 College of Medicine, The Ohio State University, Columbus, OH, United States; 4 College of Nursing, The Ohio State University, Columbus, OH, United States; 5 Grant Morrow III MD Medical Library, Nationwide Children's Hospital, Columbus, OH, United States

**Keywords:** social determinants of health, digital health, literature review, unmet social needs, health equity, public health informatics

## Abstract

**Background:**

Social determinants of health strongly influence clinical outcomes. Social needs are the individual-level, actionable facets of the broader social determinants of health framework, including food security, stable housing, and access to essential services. When these needs go unmet, they adversely affect well-being and quality of care. Systematically detecting social needs is therefore critical, and emerging digital tools now offer efficient, scalable approaches for screening and identification.

**Objective:**

This scoping review aimed to examine the use of digital health technology (DHT) or DHT-based interventions documented for screening and identifying unmet social needs in populations with high needs. We explore trends, effects, challenges, and limitations associated with these technologies.

**Methods:**

Following PRISMA-ScR (Preferred Reporting Items for Systematic Reviews and Meta-Analyses extension for Scoping Reviews) guidelines, we searched databases including MEDLINE, Embase, Scopus, ACM Digital Library, and Web of Science for studies published from 2010 to 2025. Eligible studies used technology to screen for and identify unmet social needs in populations with health and socioeconomic challenges. Data extraction focused on the types of technology, screening processes, and social needs identified.

**Results:**

Our findings highlight a limited yet evolving landscape of technological applications. We identified 14 studies using tools such as self-assessment surveys, tablet-based systems, and electronic portals. These tools were applied across diverse groups, such as refugees and patients in emergency departments. Innovative approaches, such as chatbots and multidimensional risk appraisal systems for older adults, showed potential. However, challenges included single-site studies, small samples, and integration issues with medical records. The effectiveness of these tools in screening for unmet social needs shows mixed outcomes.

**Conclusions:**

DHTs play a pivotal role in improving the identification of unmet social needs. The findings underscore the need for broader, more integrated research to fully understand the impact of technology-based assessments and screening processes for social needs. Future efforts should focus on facilitated screening using technology both within and outside of the visit, ensuring the linkage to appropriate resources and care.

## Introduction

Social determinants of health (SDOH) refer to the broad, systemic conditions in which people are born, grow, live, work, and age that shape their overall health and well-being [1]. These determinants include factors such as socioeconomic status, education, neighborhood and physical environment, employment, social support networks, and access to health care services [2-5]. Within this broader framework, unmet social needs (also referred to as health-related social needs) represent the specific, tangible challenges individuals face when these social conditions are unfavorable or insufficient. Examples include food insecurity, housing instability, transportation barriers, and limited access to essential services. These unmet needs are actionable manifestations of adverse SDOH and have a direct, measurable impact on individual health outcomes [6]. For instance, individuals experiencing food insecurity are at greater risk of chronic conditions such as diabetes, heart disease, and obesity [7]. Similarly, housing instability has been associated with an increased risk of mental health disorders, substance use, and infectious diseases [8-10]. Inadequate access to essential services, such as transportation, childcare, and health care, can further compound these health challenges [10-12].

Identifying these unmet social needs is imperative to enhancing overall health and well-being, particularly among historically marginalized populations experiencing significant health and socioeconomic challenges [13]. Although identifying unmet social needs is recognized as important, integrating screening into routine health care remains complex and often poorly implemented [3,14]. Unlike social workers and community health workers who specialize in social needs assessment, health care providers, such as physicians and advanced practice nurses, often lack the dedicated training, time, and resources necessary to effectively assess unmet social needs during clinical encounters. These health care providers may find it challenging to incorporate comprehensive screening into their already demanding workloads. This lack of capacity hampers the early identification of unmet social needs, which is crucial for informing patient care and connecting individuals to appropriate support services [15].

Traditional paper-based screening methods have struggled due to issues with scalability, documentation, resource demands, and inefficient follow-ups [16]. In contrast, the rise of digital health technologies (DHTs) provides opportunities to overcome barriers in screening for unmet social needs. Digital tools, including electronic health records (EHRs), mobile apps, and web-based platforms, offer scalable and efficient solutions for identifying and detecting unmet social needs [17,18]. Integrating social needs**–**related data into EHRs allows health care providers to track changes in patients’ social needs and better understand their circumstances, leading to more personalized care plans [18]. These technologies facilitate the systematic collection, documentation, and analysis of social needs data, enhance patient engagement, and improve referral processes to community resources**—**important aspects that are also in line with alternative payment- or value-based care model structures [19-21]. Furthermore, DHT can extend support beyond clinical settings by helping patients and families connect with essential resources, thereby ensuring continuous support and enhancing overall well-being [22,23]. However, current research on DHT-based support and assessment or studies on DHT use for screening and detecting unmet social needs are limited [24]. In addition, literature reviews reflect either the prevalence of social needs or methods to address those needs, without specific focus on DHTs [25,26].

The purpose of this scoping literature review was to (1) examine types of DHT interventions that have been documented in the literature to screen for and identify unmet social needs among populations facing health and socioeconomic challenges and (2) explore trends, effects, challenges, and limitations reported in the literature regarding the use of DHT for identifying and addressing these needs. Finally, we synthesized the findings to provide an overview of the current landscape and implementations and identified common challenges and limitations. By elucidating the role of technology in identifying unmet social needs, this study aimed to inform future research and clinical practice, ultimately contributing to the development of more effective and equitable health care assessment.

## Methods

This scoping review follows the PRISMA-ScR (Preferred Reporting Items for Systematic Reviews and Meta-Analyses extension for Scoping Reviews) guidelines (Checklist 1) [27].

### Eligibility Criteria

Within the scope of this review, the studies were selected based on 4 properties (Textbox 1) following the PICO (population, intervention, comparison, and outcomes) guidelines [28].

Eligibility criteria of this review included studies (1) with prospectively collected data, (2) that reported social need components, (3) that demonstrated a DHT-based screening method, (4) published in English, and (5) published between 2010 and 2025. This review did not include conference abstracts, poster presentations, thesis or dissertations, systematic reviews, literature reviews or meta-analyses, protocol papers, curricula, or publications in non**–**peer-reviewed journals.

Textbox 1. **PICO (population, intervention, comparison, and outcomes) guidelines.**

- Population: individuals prospectively reported 1 or more social needs. This includes, but is not limited to, people experiencing social determinants of health issues, socioeconomic factors, health service needs and demands, food insecurity, hunger, or housing insecurity.
- Intervention: use of technology for screening social needs. This includes various forms of technology such as tablets, iPads, mobile apps, chatbots, kiosks, computers, laptops, and patient portals.
- Comparison: no comparison required, but it could involve comparing technological methods of screening for social needs with traditional, nontechnological methods, or it could involve comparing different types of technological assessments or interventions with each other.
- Outcomes: the outcomes of interest could include the effectiveness of technology in identifying social needs, user satisfaction with the screening tools, integration into clinical workflows, usability, and any challenges encountered during the screening process.

### Search Strategy

**Figure 1.**
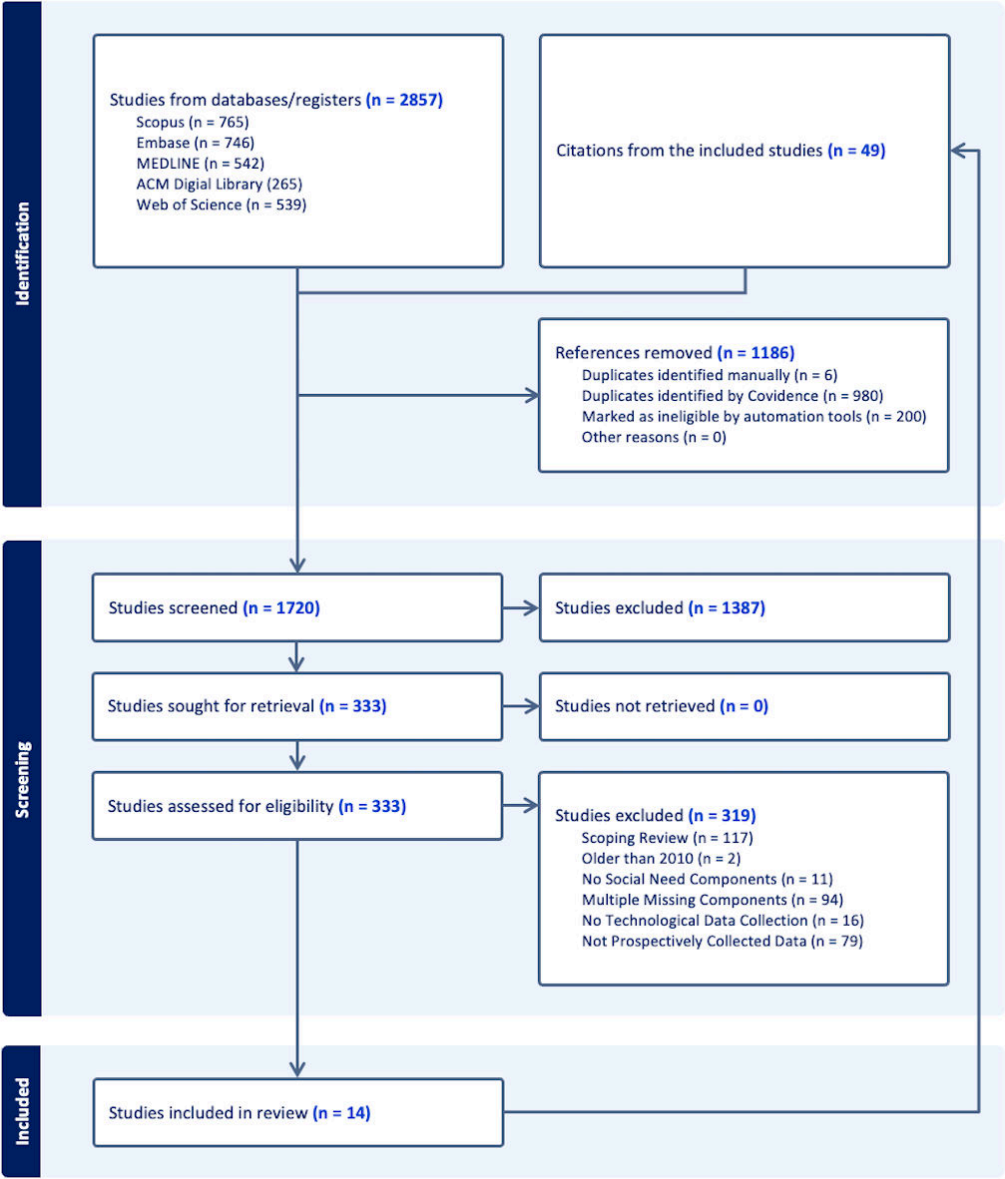
PRISMA-ScR (Preferred Reporting Items for Systematic Reviews and Meta-Analyses extension for Scoping Reviews) diagram.

We used scientific peer-reviewed academic literature databases including MEDLINE, PubMed, Embase, Scopus, ACM Digital Library, and Web of Science (Figure 1). The search was conducted on May 20, 2025. We extracted 2857 peer-reviewed articles using the Covidence software (Veritas Health Innovation Ltd; EL) [29]. The researchers (ES, DM, EL, and DIJ) developed the database search queries using 3 groups of keywords: social needs, data collection approach, and technology used. Multimedia Appendix 1 outlines the search query. Additional literature (49/448, 10.9%) was included via searching and reviewing citations from the included papers (14/2857, 0.05%).

### Data Extraction

Articles included in the search were cataloged and systematically reviewed to be included based on the PICO guidelines (EL, DIJ, BF, and ES). Duplicate studies were removed automatically (1180/2857, 41.3%) by Covidence and manually (6/2857, 0.2%). Two researchers (DIJ and BF) screened the titles and abstracts of the remaining studies (1720/2857, 60.2%) with cross-verification and supervision by the senior researcher (ES). Conflicting reviews were resolved by consensus after an open discussion among the team members. Studies were assessed for full eligibility (333/1720, 19.3%), and 95.8% (319/333) were excluded for various reasons such as missing social need components, use of technological data collection, or a retrospective study design. In total, 0.4% (14/2906) of studies were included for full eligibility in the review and were extracted (ES, SB, DIJ, LM, MS, and MD) by categorizing the study design, demographics, findings, and other characteristics (Multimedia Appendix 2 [30-43]).

## Results

### Demographics

The included studies were primarily conducted in the United States, with sample sizes for participants ranging from 13 to 826 (Table 1). Social needs were most frequently assessed in the English language (13/14, 93%) and were about food insecurity (10/14, 71%) and housing instability (9/14, 64%). Participants were predominantly female (10/30, 33.3%-528/748, 70.6%) and White (6/154, 3.9%-370/430, 86.1%), with notable representation from Black and African American (8/78, 10.3%‐324/507, 63.9%) and Hispanic/Latinx (15/254, 5.9%‐53/101, 52.4%) populations. Tablet PC surveys were the most common DHT used (6/14, 43%), while other DHTs such as chatbots, software and web apps, and text messaging services were less frequently used (2/14, 14% each). Most studies (11/14, 79%) were published between 2019 and 2023. The most common study outcome reported was the ratio of total patients screened. Participants varied widely in age, race, ethnicity, and socioeconomic backgrounds.

**Table 1.** Characteristics of the included studies (N=14).

Characteristic/category		Values
Study locations^a^, n (%)		
	United States	10 (71)
	Spain	1 (7)
	United Kingdom	1 (7)
	Canada	2 (14)
Sample size ranges^b^, N		
	Patients	13‐826
	Caregivers	30‐505
	Health care providers	13‐147
Languages^b^, n (%)		
	English	13 (93)
	Spanish	6 (43)
	Farsi/Dari	1 (7)
Social needs assessed, n (%)		
	Food insecurity	10 (71)
	Housing instability	9 (64)
	Health care accessibility	8 (57)
	Transportation	7 (50)
	Childcare/education	6 (43)
	Financial stability	6 (43)
	Exposure to violence	3 (21)
Sex/gender identity, % (range)		
	Female	10‐528 (33.3‐70.6)
	Male	17‐296 (29.4‐56.7)
	Other	3‐16 (10.0)
Race/ethnicity of participants, % (range)		
	White	8‐324 (3.9‐86.1)
	Black/African American	15‐53 (10.3‐63.9)
	Hispanic/Latino/Latina/Latinx	6‐370 (5.9‐52.4)
	Multiple or other	7‐85 (0.5‐33.4)
Types of technology, n (%)		
	Tablet survey	6 (43)
	Electronic health record portal	2 (14)
	Software/web apps	2 (14)
	Chatbots	2 (14)
	Text messaging	2 (14)
Study outcomes, n (%)		
	Percentage of patients screened	11 (79)
	Patient satisfaction	6 (43)
	Patients referred to consultation	6 (43)
	Number of health care visits	5 (36)
	Patient health literacy	4 (29)
	Patient screening task load	3 (21)
Publication year, n (%)		
	2024	1 (7.14)
	2023	3 (21.4)
	2022	2 (14.3)
	2021	2 (14.3)
	2020	2 (14.3)
	2019	2 (14.3)
	2017	1 (7.14)
	2012	1 (7.14)

a Some studies were multisite.

b Sample sizes are not mutually exclusive; subsamples derived from a larger sample may overlap.

### Types of DHTs

Tablet-based computers [30,31] and EHR-based tools were most commonly used technologies among the studies within the scope [32,33]. These technologies were used to facilitate the screening, recording, and referral process for unmet social needs within health care settings. Web-based systems and electronic portals were also used, such as an online screening tool [34] or the electronic portal via REDCap (Research Electronic Data Capture; Vanderbilt University; online survey platform) integrated with the 2-1-1 services [35] Other DHTs included cross-platform apps with Health Insurance Portability and Accountability Act (HIPAA)–compliant cloud infrastructure for data collection and analysis [36] and a software designed for multidimensional risk appraisal in older adults [37]. In addition, text message–based services [38] were used for social needs screening and were linked to REDCap surveys [39]. Similarly, 2 studies explored text-based screening via innovative approaches such as chatbots [40,41], which leveraged natural language processing to engage users and enhance data collection.

### Unmet Social Needs Assessment

As presented in Table 1, the studies examined various social needs. More specifically, these included psychosocial risks for refugees [30], caregiver needs in pediatric inpatient units [42], financial needs [32,34,38], food insecurity [31,32,38,42] in primary care settings, and general social needs in diverse patient populations [34,41]. Housing instability and financial strain were also screened in primary care and pediatric settings [42,43]. Furthermore, environmental, psychosocial, and behavioral factors were reported toward reducing cardiovascular risks in African American young adults [36]. During the COVID-19 pandemic, unmet social needs were evaluated in urban emergency departments [39].

### Research Methods and Approaches

Common study designs included mixed methods and single-arm studies [31,34,35,38,41], as well as quasi-experimental designs [33,39]. Two studies assessed feasibility through simulated clinical workflow implementations [35,37]. Three studies were reported as quality improvement projects [35,42,43], and 1 study was a pilot randomized controlled trial [30]. Two quality improvement studies used “plan-do-study-act” cycles for rapid prototyping and adapting the study intervention based on stakeholder (eg, caregiver, health care provider, and patient) feedback longitudinally [42,43]. Two other studies used implementation science frameworks, specifically reach, effectiveness, adoption, implementation, and maintenance (RE-AIM), to evaluate the feasibility of screening technologies [31,38]. In addition, other approaches involved community-based participatory research and user-centered design for mobile platforms [36], within-subjects designs for survey comparisons [40], and validation studies [32]. Studies used a variety of scales for measuring SDOH [33,38,40,41], unmet social needs [30,31,35,37-42], usability [33,36,38,40], and postintervention cost-effectiveness [37]. However, no overlapping measures or consensus on outcome metrics, such as the effects of screening or DHT use, were observed across the studies.

### Opportunities and Challenges

The studies reported a number of opportunities and challenges associated with using DHT to identify unmet social needs. In particular cases, patients may express more comfort seeking care services through DHT-based platforms. For example, Ahmad et al [30] reported that 72% of participants who used a touch screen self-assessment intended to seek psychosocial counseling, compared to 46% in usual care. Commonly identified needs included mental health support, food security, and access to public utilities, with 1 study noting that 99% of family caregivers received contact information and details for federal and community-based aid programs [42]. Another study indicated that participants who self-reported unmet social needs via the DHT-based screening tools had high follow-up rates with local support programs after clinical visits (as high as 86%), leading to sustained patient engagement throughout study procedures [37]. A tablet-based screening approach reported an increase in screening rates (from 45% to 90%) [43]. However, the use of DHT did not eliminate the challenges of documentation and management of positive screenings, such as missing documents needed to receive community resources [43]. Conversational DHTs, such as chatbots, were observed to improve screening engagement and comprehension of social needs**–**related content, particularly among participants with low health literacy [40,41]. Personalization features (language options, multiple modalities, interaction timing, and location awareness) were noted as a likely contributor to the adoption of digital screening tools, especially in marginalized groups [30,34,38,39,42]. However, only text messaging for screening recruitment was deemed infeasible for minoritized populations [38,39]. The introduction of electronic methods, such as tablet-based systems, resulted in higher social need screening administration rates but introduced new concerns between the personal interactions of patients and health care providers, leading to a decrease in documentation quality in favor of operational efficiency [43].

### Key Observations

A common observation across the studies was the heterogeneity of the DHTs and tools used, such as tablet-based self-assessment systems [30,31] and electronic portals [35], to facilitate the screening and referral processes for social needs. Several studies focused on specific populations, including Afghan refugees [30], caregivers in pediatric settings [42], and patients visiting emergency departments [35], reporting the technological assessment to diverse demographic groups. Additionally, the majority of studies aimed to evaluate the feasibility and effectiveness of these technological tools in routine health care settings, focusing on practical implementation and cost considerations [31,34,35,37,39,42]. Novel approaches and recent DHTs were observed, such as chatbots for SDOH screening [40,41] and the integration of multidimensional risk appraisal systems [37]. Studies also explored the effectiveness of these approaches in improving social needs**–**related outcomes associated with quality of life, stress, food insecurity, financial stability, housing stability, and service use [32,33,36,43]. Some of the observed outcomes included a reduced risk of cardiovascular disease and improved sleep quality [36], enhanced detection of unmet social needs [32], and the creation of a more equitable system for accessing community and health services [33,43]. Although survey-style screening tools were the most commonly used in the sample of studies (6/14, 43%), software apps and chatbots (3/14, 21%) incorporated more process measures, including patient satisfaction and successful resource referrals. DHT interventions that demonstrated notable improvements in addressing social needs and broader SDOH were those that incorporated patient feedback and supported customization or self-reflection, such as tailored recommendations or risk summaries, thereby enhancing perceived usefulness by improving understanding and engagement among users with lower health literacy, increasing intention to seek counseling, boosting multilingual referrals to services, and raising awareness of behavior-related risks in minoritized populations [30,35,36,40].

### Limited Applications

Common limitations included single-site studies and small sample sizes, affecting generalizability [30,31,38,42,43]. In addition to that, studies reported the potential self-selection bias and short study durations, impacting the assessment of long-term effects [40,41]. Several studies were missing key demographic features of the study sample. This included the criteria for identifying low digital literacy in patients [34], socioeconomically disadvantaged patients [32,37], and domain-specific medical conditions [40]. Following that, Walters et al [37] reported increased inequitable access between racial and ethnic communities. In terms of DHT, Berger-Jenkins et al [43] noted DHT-related problems, such as integration challenges with existing medical records systems and documentation difficulties [44]. The inability to track resource map use or assess successful linkages to community-based organizations was noted as a barrier [42]. With text-based services, it was unknown when the screening was completed or if the content was understood well, which was noted as some of the potential limitations [39,40].

## Discussion

### Principal Findings

This scoping review was conducted to examine the use of DHT or DHT-based interventions documented for screening and identifying unmet social needs within populations with high needs. Our findings reveal a limited but evolving landscape of technological applications, transitioning from tablet-based surveys to more intelligent chatbots. This evolution suggests a potential shift toward facilitating communication with patients outside of clinical visits, thereby enhancing continuous support and engagement.

DHT-based screening tools have been useful for identifying unmet social needs by easing the process of screening, leading to an increased number of screenings completed. For example, the use of touch screen self-assessment surveys led to a higher number of requests for psychosocial counseling among participants compared to usual care [30]. Similar findings were observed with tablet-based or online tools toward identifying unmet social needs, such as food insecurity, utility assistance, and mental health needs [31,42]. This contributes to the use of DHT in social services and is consistent with the broad digital health transformation process [45]. However, using DHTs for large-scale screening may require a broad adoption strategy supported by organizational or cross-institutional leadership to ensure long-term effectiveness [46]. A federally qualified health center investigated patient perceptions of patient portals and reported high levels of access and use among patients**—**75% of patients rated the DHT as highly useful as an administrative aid after institutional deployment [47].

For sensitive topics, some patients may feel more comfortable self-reporting through DHT, thereby avoiding direct human interaction [48]. The current literature has identified certain trends that alter a user’s willingness to disclose to diagnostic chatbots and similar automated services [49-51]. This design philosophy around health-sensitive information may require further investigation to understand its long-term effects with regard to the end user. Furthermore, integration of innovative technologies such as chatbots may show increased engagement or support comprehension of social needs and promote interaction with community services [52]. Particularly among individuals with low health literacy, it can help contextualize the needs [40,41], further promoting behavioral interventions and health information communications [52-54]. Toward that direction, research institutions and community health centers have focused on community-based participatory research or user-centered design as foundational principles to further adapt tools for patient populations at risk that struggle with health literacy [30,33,36,41].

The effectiveness of screening with DHTs presented mixed outcomes. While tablet-based systems demonstrated an increase in screening completion rates and higher engagement in consultation, the long-term impact remained unclear. The findings were limited to indicate sustained effects of identified unmet social needs. For instance, while an online database helped connect patients with 2-1-1 information specialists within a week of leaving the emergency room [35] and access to new community resources increased after screening positive for an unmet social need [31], more patients followed up for social worker consultations [42]. This showed a short-term success in practice, but organizational implementation and sustainability of such screenings in the long term remained challenging [55-57]. Integration of DHTs with existing systems (eg, medical records and referral registries) posed technical and workflow barriers [58], and documentation gaps hindered continuity of care [43,59]. Additionally, limited health care provider buy-in, lack of system interoperability, and unresolved data privacy concerns were rarely addressed but are critical barriers. Overcoming these challenges may require early stakeholder engagement, alignment with clinical workflows, and clear data governance strategies to ensure adoption and sustainability. These implementation problems might not be unique, as they have also been reported consistently in a number of studies on adoption of DHTs [60-62].

Additionally, the low recruitment rates and the limited feasibility observed with the selected DHT platforms emphasize the importance of context-specific strategies and DHT selections tailored to the target population’s needs and preferences [37-39]. For instance, Pratap et al [63] reported that the response and retention to DHTs may show differences based on the target population. The demographic differences observed in DHT adoption (eg, age, race, and literacy) suggest that while DHT can enhance access to care, it can also exacerbate existing inequities if not carefully designed and implemented [37]. Therefore, we must consider the barriers specific to different demographic groups to ensure equitable access to health resources. Including diverse teams and stakeholders in the development and deployment of DHT to assess social needs is an important way to ensure the wide reach and use of DHT in the health care setting [64].

### Comparison With Prior Literature Reviews

Previous reviews focusing on DHT use, SDOH, and unmet social needs identified themes similar to those observed in our study [65-67]. Maaß et al [66] reviewed digital health interventions from a public health perspective. Their work focused on mapping each intervention based on the size of regional application (ie, proposed, pilot, national, and international). Our review expands on the heterogeneity of digital health applications and reports recent and novel screening tools that were adopted for different settings and populations. Yao et al [65] highlighted the inequities of digital health studies when implementation practices do not focus on health disparities and instead exacerbate the impact of inaccessible technological tools. Similar to our findings, the authors identified a patient’s socioeconomic status as a major underlying factor to these disparities, as not all patients, visitors, and stakeholders have equal access to the DHTs. Finally, Craig et al [67] reported a group of studies under 3 categories in digital health: policy, data, and technology. From a public health perspective, they reported that behavior-related SDOH assessments and tailored interventions (ie, a weight loss program [68]) may lead to a greater improvement of health outcomes.

### Limitations and Future Works

The search strategy was confined to the selected databases and only included studies published in English, potentially introducing selection bias. Studies were not regionally restricted to the United States, but the terminology used may have resulted in a higher proportion of studies from the United States. As the majority of the studies were conducted within the United States, it should be noted that the lack of global representation can affect the generalizability, particularly for non–English-speaking and low-resource settings. Nonetheless, we included studies that investigated or supported multiple languages, alongside English. Keywords in our search were limited to SDOH, social needs, and social risks, not including subcategories or contributing factors to SDOH (eg, food insecurity or economic instability). The heterogeneity of the assessment approaches and outcomes in the included studies, along with the inclusion criteria, may limit the comprehensiveness and comparability of the findings. The variable quality of evidence, with some studies having methodological limitations, further constrained the ability to draw definitive conclusions. We did not perform a meta-analysis or risk of bias assessment due to the nature of scoping review and the variability in study designs, populations, interventions, and outcomes, which limits the ability to quantify the overall effect of DHT-based assessments.

Future research should include additional and diverse databases and non-English studies, explore longitudinal methods to assess screening impact, and develop standardized outcome measures to improve the breadth and comparability of reviews. On the basis of the heterogeneity of study designs within our scope, we recommend that future research projects provide systematic reporting of patient-reported outcomes, following guidelines such as CONSORT-PRO (Consolidated Standards of Reporting Trials for Patient-Reported Outcomes extension), STROBE (Strengthening the Reporting of Observational studies in Epidemiology), or SQUIRE 2.0 (Standards for Quality Improvement Reporting Excellence). Including the measures to assess how unmet social needs are addressed and conducting comparative cost-effectiveness analyses can provide more practical insight. Additionally, patient-centered research to understand user experiences and preferences can guide the development of more acceptable and effective screening tools.

### Managerial and Practical Implications

The findings of this scoping review present several critical implications for key stakeholders involved in addressing unmet social needs through DHTs. Policymakers must recognize the current scarcity of research on DHT-based interventions for social needs screening and prioritize efforts with policies that support the development and evaluation of innovative digital tools. This could promote equitable access and design to prevent exacerbation of existing health disparities. Clinical informaticians are essential in advancing DHTs, focusing on integration with EHRs and enhancing user experience to facilitate ongoing patient–health care provider communication outside of clinical visits. Furthermore, social workers, nurses, care coordinators, and other acute health care teams could leverage these DHTs to streamline referral processes and maintain continuous engagement with clients, thereby enhancing support after discharge.

Additionally, health care providers may require training and support to effectively implement these tools within clinical workflows, while health care administrators may need to allocate resources and facilitate integration with existing systems. Therefore, engaging with health care providers, community partners, patients, and caregivers in the design and implementation process ensures that the DHTs meet their needs and preferences, enhancing usability and effectiveness. Collaborative efforts among these stakeholders, including technology developers, vendors, and researchers, are essential to bridge existing research gaps, optimize the implementation of technological solutions, and ultimately improve health outcomes to achieve greater health equity.

### Conclusions

This work highlighted the potential of DHT used for identifying unmet social needs across diverse populations. The reviewed studies demonstrated the use of various DHTs for enhancing the screening and referral processes for social needs. However, DHTs also presented challenges that could potentially exacerbate existing health disparities if not thoughtfully implemented. To fully realize the benefits of DHT-based screening, it is crucial to drive the field toward studying the complete context of the screening process. This includes not only the initial identification of unmet social needs but also the follow-up actions, reception of services, and the subsequent improvement of health and social outcomes. We suggest more research on comprehensive evaluations that track the care continuum from screening to outcomes, ensuring that DHT-based solutions lead to meaningful and sustained improvements in addressing unmet social needs.

## Supplementary material

10.2196/78793 Multimedia Appendix 1 Search query.

10.2196/78793 Multimedia Appendix 2 Literature search results.

10.2196/78793 Checklist 1 PRISMA-ScR checklist.

## Abbreviations

CONSORT-PRO: Consolidated Standards of Reporting Trials for Patient-Reported Outcomes
DHT: digital health technology
EHR: electronic health record
HIPAA: Health Insurance Portability and Accountability Act
PICO: population, intervention, comparison, and outcomes
PRISMA-ScR: Preferred Reporting Items for Systematic Reviews and Meta-Analyses extension for Scoping Reviews
RE-AIM: reach, effectiveness, adoption, implementation, and maintenance
REDCap: Research Electronic Data Capture
SDOH: social determinants of health
SQUIRE 2.0: Standards for Quality Improvement Reporting Excellence
STROBE: Strengthening the Reporting of Observational Studies in Epidemiology
