# Supplementary material for: Digital Health Technologies for Screening and Identifying Unmet Social Needs: Scoping Review
Source: J Med Internet Res. 2025 Nov 11;27:e78793. doi: 10.2196/78793 (PMC12604432; doi:10.2196/78793)
Supplement: Multimedia Appendix 2 [file jmir-v27-e78793-s002.docx]

| **Study** | **Study purpose** | **Technology & Intervention** | **Sample size and location** | **Research methodology & Instruments** | **Analysis** | **Major findings** | **Strengths & Limitations** | **Eligibility criteria reported** |
| --- | --- | --- | --- | --- | --- | --- | --- | --- |
| Ahmad et al. 2012 | The primary objective was to examine the potential of a computer assisted psychosocial risk assessment for refugees and the integration of medical and social services. | A touch-screen self-assessment survey completed on a touch-screen iPad in Dari/Farsi language while waiting to see a healthcare provider. It was a computer-assisted psychosocial risk assessment (CaPRA) survey | Afghan refugee patients > 18 years of age; speak and read Dari/Farsi or English, eligible for federal or provincial health care and visiting a participating provider; N= 64 eligible; 50 participants. Location: Toronto, Canada - Single Site | Pilot randomized control trial.  Instruments:  Patient Intention Computerized Lifestyle Assessment Scale (CLAS) Patient Satisfaction | Descriptive statistics; chi-square and t-test for two group comparisons. Some response categories were collapsed due to small sample size | 72% of participants in the CaPRA group intended to visit a psychosocial counselor vs. 46% in usual care. CaPRA group participants agreed with the benefits of the tool (mean=4 on 5 point scale) but were unsure about barriers to interacting with clinicians or privacy info (mean =2.8). Both groups were alike in satisfaction (mean = 4.3). | Strengths include the randomized control trial design, international scope, and multilingual capabilities. Limitations include being a single-site study with a limited sample size, which may affect generalizability, as well as demographic differences between groups. | Afghan refugee patients > 18 years of age; speak and read Dari/Farsi or English, eligible for federal or provincial health care and visiting a participating provider |
| Fortin et al. 2021 | To develop and test the feasibility of a caregiver self-administered social needs screener, a Web-based searchable community resource map, and a process map for implementation of these tools as part of social needs screening and referral on a pediatric inpatient unit. | The screening tool was programmed as a Research Electronic Data Capture (REDCap) survey and deployed by using electronic tablets. The resource map is a web site database of community-based organizations (CBOs) searchable by social need category and geographic location. Search results can be filtered by programmatic characteristics and preferences such as language spoken and hours of operation. EHR quality improvements access to intervention tools, and workflow for implementation. | Caregivers, care team assistants; 145 caregivers participated.  Location: Pennsylvania, US - Single Site | Quality improvement project.  Instruments: Patient Health Questionnaire-2 | Descriptives, PDSA cycles and run charts | Thirty-four percent of caregivers endorsed $1 social need. The most common needs identified were depressive symptoms (23%), food insecurity (19%), and need for assistance with utilities (10%). All participants received information about the resource map, and 99% of caregivers with an identified need met with a social worker during their admission. | Strengths include the strong use of QI methodology with multiple PDSA cycles and a well-designed process map, along with the implementation of a web-based resource map. Limitations include the inability to track the number of resource map searches directly attributable to caregivers in the project unit and the lack of assessment on whether families successfully linked to community-based organization (CBO) referrals, as well as the single-site study limiting generalizability. | None reported |
| Palakshappa et al. 2021 | To determine the feasibility of using a mobile health system to screen for patients’ social needs. | A tablet-based digital health system was developed to address patient's unmet social needs (using questions from the CMS AHC Health-Related Social Needs Questionnaire). Once screened, the system automatically provided a list of community resources in the patients' after visit summary if the patient indicated an unmet need (e.g. food pantries, organizations that assist with housing) in addition to notifying the provider that the patient had screened positive - if available a "navigator" would meet with the patient at the time of the visit. | All adult patients (>18 years) that speak either English or Spanish, and presenting for a non-urgent visit at a primary care clinic in Wake Forest Baptist Health were eligible. 219 patients completed the tablet survey. 23 providers completed the acceptability survey.  Location: North Carolina, US - Single Site | A single-arm investigative study was conducted to assess the feasibility, acceptability, and efficacy (1 month follow-up)  Instruments:  Reach, Effectiveness, Adoption, Implementation, Maintenance (RE-AIM) System Usability Scale (SUS) Provider Acceptability Survey  CMS Accountable Health Communities Health-Related Social Needs Screening Tool | Bivariate analyses, chi square tests, and multivariate logistic regressions were used to test associations among covariates and whether the patient reported an unmet need and between covariates and those who reported accessing at least 1 community resource. | Overall, the study found the mobile health system to screen patients as feasible and acceptable (however, 20% of the patients required assistance with the tablet, such as help with entering responses). Approximately two thirds (68.5%, n = 150) of the participants screened positive for at least 1 unmet social need (116 [53.0%] for food insecurity, 86 [39.3%] for housing problems, and 74 [33.8%] for lack of transportation). | Strengths: RE-AIM framework was used to guide and to assess the outcomes in the study. Both English and Spanish speaking populations could use it. Different types of providers were included in the assessment (physicians, advanced practice providers, nurses, and other staff members). Detailed tracking of study flow diagram. Limitations: This study is derived from one single study site, which hampers generalizability and concurrent state-wide use of NCCARE360, a statewide resource platform to allow for electronic referrals to community resources. In addition, it is unclear which intervention increased patients' access to resources (e.g. the paper-based list of resources or the meeting with the patient navigator). | All adult patients (>18 years) that speak either English or Spanish, and presenting for a non-urgent visit at a primary care clinic in Wake Forest Baptist Health were eligible. |
| Pinto et al. 2019 | To assess the acceptability and feasibility of integrating an online tool to help identify financial benefits in primary care. | An online, web-based tool developed in collaboration with Prosper Canada (a national Canadian charity) and with input from community organizations and community health care center staff. The study assessed the acceptability and feasibility of providers screening their patients for poverty using an online tool that also identifies financial benefits for which the patients could be eligible. | 13 physicians were asked to use the online tool with as many patients as possible between 23 September 2015 and 23 October 2015 (N = 63 patients). Eligible patients were recruited by 13 providers during 1 month in 2015 at the Community Health Centre.  Location: Toronto, Canada - Single Site | A mixed-methods study - which included physician and participant feedback via surveys and focus groups (e.g. on use of the tool, perceived value of physician-led screening, physician confidence on identifying and suggesting resources, perceived value of the tool).  Instruments:  perceived value survey | Descriptive analyses of survey responses data, and open-ended responses were organized thematically using content analysis procedures. | A low number of physicians reported using the tool during patient encounters over the study duration, with each physician, on average, using the tool with fewer than five patients (despite being asked to use it with every patient seen). Overall, physicians found the screener to be a worthwhile tool (with some noting they learned more about their patients' lives), which could be modified to be more effective (especially for non-English speaking patients). | Strengths include the inclusion of community and staff input in designing the online tool and allowing physicians the choice in administering the tool, which was targeted to either new patients or those with financial assistance needs identified during the appointment. Limitations include the lack of a clear definition for "low-income," a low sample size for both physicians and participants, limited use of the tool during the study period, and the focus on a single primary care organization, which restricts generalizability. | Limited eligibility criteria included. Individuals attending one clinic during one month in 2015, and physicians made the decision on whether or not the participant would be included. |
| Wallace et al. 2020 | To develop and evaluate a process for systematically identifying social needs during routine health service delivery, for facilitating access to community-based supportive services, and for integrating existing clinical (ie, Epic) and community-based referral data systems. | The study used an electronic portal integrated with REDCap to export social needs screening results to the 2-1-1 system for community service referrals, with data stored in the Epic data warehouse. The intervention included: 1) developing a streamlined social needs assessment, referral, and evaluation process; 2) assessing the feasibility of implementing this process during routine emergency department care delivery; and 3) evaluating the nature, quality, and utility of linking social needs assessment data with the 2-1-1 referral database and selected fields from electronic health records (EHRs). | 210 ED patients at a hospital in Utah for four one-week time periods in 2017 and 2018. Collected 210 patient responses. Location: Utah, USA - Single Site | 2-phase, mixed-methods feasibility study with  evidence-based organizational improvement model  Instruments:  Social Needs Screening Toolkit | Wilcoxon signed rank test was used to examine the number of visits 3 months before versus 3 months after the emergency department index date. The analyses compared 1) patients who expressed at least 1 need (n = 107) and patients with no reported needs (n = 55); and 2) patients whom 2-1-1 attempted to contact, those who received 2-1-1 services (n = 32), and those who did not receive 2-1-1 services (n = 33). | Service use 3 months before versus 3 months after the emergency department index date show that patients with at least 1 social need had a significant increase in emergency department use (1.07 before vs 1.36 after, P = .03) while patients with no needs had an increase in primary care visits (0.24 before vs 0.56 after, P = .03) (Table 2). The trend of increased emergency department visits was also noted among those who received follow-up and referrals from 2-1-1 (1.97 before vs 2.56 after, P = .006) (Table 3). We found no differences in hospitalizations between the 2 groups. | Strengths include the availability of the screening tool in Spanish and its demonstrated ability to systematically screen and refer emergency department patients for unmet social needs. Limitations include ongoing concerns from ED team members about who should conduct the screenings, discomfort in asking potentially stigmatizing questions, and patients questioning the purpose of the screening. Although some team members expressed interest in allowing patients to self-complete the screening, observations showed that this was rarely implemented in practice. | Patients in the ED who were screened for social needs over 25 days between Nov 2017 and July 2018 |
| Walters et al. 2017 | The aims of this study were to test the feasibility and costs of using Health Risk Appraisal for Older people (HRA-O) and Smarter Working in Social and Health Care project (SWISH) tools combined into a Multi-dimensional Risk Appraisal system for Older people (MRA-O), including local embedding and re-enforcement of use in routine primary care. | The Multi-dimensional Risk Appraisal for Older people (MRA-O) system, a computer based software, was used. Intervention stages included: 1) tailoring the MRA-O system to local needs, systems and services. 2) conducting assessments using postal questionnaires with a random sample of older people registered with participating General Practices. Data were entered and automated personalized feedback was provided to individuals . A copy of the feedback report was scanned into participating patients’ GP health records and reviewed by a lead clinician in each practice. | Total participants = 454 (171 Urban (Ealing) and 283 Semi-Rural (Hertfordshire)), living in two localities in the UK representing urban and semi-rural locations, with different proportions of black and minority ethnic (BME) populations and different socio-economic characteristics. Location: London and Hertfordshire, UK - Multisite (5) | Feasibility study  Instruments:  UK 2011 Census,  Multi-dimensional Risk Appraisal for Older people (MRA-O) | Data from the MRA-O software were generated as Excel files and transferred to SPSS. Other data were directly entered in SPSS. Data were double entered and discrepancies resolved. Data were cross-checked by tabulations and histograms identifying missing data and anomalies and cleaned with reference to the original questionnaires. Descriptive data analysis was undertaken only, as the primary purpose of this study was to determine the feasibility of implementing this process. | Response rates to initial postal invitations were low (526/1550, 34%). Of these, 454/526 (86%) completed MRA-O assessments. Practices reviewed medical records of 143/454 (31.5%) of participants as a consequence of their responses, and actively followed up 110/454 (24.2%) of their patients. | Strengths include the novelty of the system, which generates personalized reports on both health and social concerns, and its successful integration into routine primary care as a feasible case-finding tool for identifying health and social needs among older adults. Limitations include higher usage among owner-occupiers, white ethnic groups, and the 'younger old' age group (65–84 years), which suggests that the intervention may contribute to increased inequalities in access. | Exclusion criteria: living in nursing or residential care settings; with severe incapacitating/life-threatening/terminal illness; unable to provide informed consent (e.g. with moderate to severe dementia/learning disability); where an assessment would be considered burdensome at the time of recruitment (e.g. very recent bereavement or life-threatening illness). |
| Clifford et al. 2022 | To develop a system design for the MOYO mobile data collection platform using the design requirements for an urban African American population. The function of the MOYO mobile platform is to gather longitudinal and granular environmental, psychosocial, behavioral, and clinical data from a population of young African American adults to reduce CVD risk factors. | MOYO mobile platform, a cross-platform app to collect standardized health surveys, narratives, geolocated pollution, weather, food desert exposure data, physical activity, social networks, and physiology through point-of-care devices. A HIPAA–compliant cloud infrastructure was developed to collect, process, and review data, as well as generate alerts to allow automated signal processing and machine learning on the data to produce critical alerts. Integration with wearables and electronic health records via fast healthcare interoperability resources was implemented. | Urban African Americans, aged 18 to 29 years (n=505)  Location: Georgia, USA - Multisite (3) | Electronic cohort study; CBPR + User centered design via co-design sessions to ensure engagement & usability of MOYO.  Instruments:  Patient Health Questionnaire-9 Kansas City Cardiomyopathy Questionnaire-12 Patient-Reported Outcomes Measurement Information System Global-10 Quality of life, enjoyment, and satisfaction questionnaire Subjective Units of Distress Scale Posttraumatic Stress Disorder Checklist Mood Zoom Mood Swipe | multivariable logistic regression. Additionally, bivariate analyses | A total of 9 themes (customization, incentive motivation, social engagement, awareness, education, or recommendations, behavior tracking, location services, access to health professionals, data user agreements, and health assessment) were identified from 297 prototype screens as images; elements that emerged can be organized into 4 overarching categories: individual, interpersonal, expert-informed, and technological; gamification and use of behavioral economics are perhaps keys to the retention of users | Strengths include the use of a CBPR and UCD design approach, along with a cloud-based platform that allows for automated and scalable analysis. Limitations include the integration with wearables, which has been demonstrated through a proof of concept, but raises concerns about the utility of crowdsourcing quasi-medical data without standardization. Additionally, firmware or device updates mid-study could disrupt data collection. | Inclusion criteria were (1) self-identification as Black or African American, (2) aged between 18 and 29 years, and (3) own an iPhone operating system or Android-based smartphone. |
| Dommaraju et al. 2022 | The objective of this work was to implement technology-based enrollment and patient engagement and reflect on the challenges encountered when using this method to conduct social needs assessment of patients presenting with COVID-19-like illness at an urban academic emergency department. | An online survey platform REDCap (Research Electronic Data Capture) was used, through which a standardized text message was sent to the mobile devices of eligible patients who tested positive for COVID-19. Patients were first provided with information on social services (e.g., health, food, transportation, housing). After e-consent, they were then asked to complete a social and health needs assessment on the first day and 14th day after COVID-19 testing. | 13 urban, academic ER with primary service area of predominantly African-American/Black and Hispanic/Latino(a) neighborhoods. Location: Illinois, US - Single Site | Quasi-experimental feasibility study  Instruments:  COVID-19 Testing Registry Brief Health Literacy Screen (3) | Multivariable logistic regression were performed | The results of the study suggest that our text-based recruitment platform is not a feasible tool to conduct a social needs assessment, in consideration of our specific study population. Most of the eligible participants who received a REDCap link via text message did not open the link and fewer consented to participate. Adding an incentive to the study did not increase enrollment. | Strengths include findings that highlight the importance of studying and improving digital access and literacy to ensure equitable health communication platforms. Limitations include the possibility that text message invitations were sent at inconvenient times or with delays of hours to days after the patient’s ED visit, the recruitment text may not have been written in language understandable or relatable to the patient population’s social context, and the REDCap platform may have been too complex, requiring patients to navigate through multiple resource sets, potentially leading to burnout before reaching the research survey. | Patients who were 18 years or older, had been tested for COVID-19, and were discharged after being tested were eligible for inclusion in the registry. |
| Harle et al. 2023 | Assessed the accuracy of EHR-based multidomain questionnaires using single-domain questionnaires on food insecurity, housing instability, and financial strain as external standards. | EPIC EHR-based tool was used.  Patients completed during visits, via phone, or email after the visit. | 826 adult patients receiving care at 1 of 11 primary care clinics in Indianapolis, Indiana and Gainesville, Florida. Location: Indiana & Florida, USA - Multisite (11) | Validation study  Instruments:  United States Department of Agriculture's Six-Item Short Form of Food Security SurveyUnited States Department of Agriculture's Housing Instability IndexConsumer Financial Protection Bureau’s Financial Well-being Scale | Compared differences in the percentage of patients with a positive screen for each social factor using a z test. In addition, they calculated sensitivity, specificity, positive predictive values, and the area under the curve (AUC). | The study found that the prevalence of food insecurity was not significantly between the EHR-based screening questionnaire and the single-domain questionnaire. There was a significant difference in the prevalence for housing instability. The prevalence from the EHR-based questionnaire was significantly lower than from the single-domain questionnaire, as was the case for financial strain as well. The EHR screening questionnaire for food insecurity had strong performance, with an AUC of 0.938, high sensitivity (94.5%) and specificity (93.1%). In contrast, housing instability had a lower AUC of 0.760, with high specificity (93.0%) but lower sensitivity (59.0%). Financial strain showed an even lower AUC of 0.626, with very high specificity (98.1%) but low sensitivity (27.1%). | Strengths include the accuracy of EHR-based food insecurity screening. Limitations include potential issues with generalizability, as the sample was drawn from only two health systems in two states. | Adults receiving care in primary clinics between January and September 2022 that completed EHR-based and single domain questionnaire concurrently in either English or Spanish. |
| Rodoreda-Pallàs et al. 2023 | Design and evaluate an intervention to increase the recording of SDHs in computerized medical records in primary care consultations in the area of Central Catalonia (Spain). | eCAP program, an electronic patient health record system was used. The intervention consisted of nine 2-hr face-to-face training sessions for providers from all professions in three primary care services. The intervention consisted of introducing uniform definitions of the social determinants of health, reviewing 18 cases that providers may encounter, and a checklist was introduced to minimize the time burden on providers deciding whether or not to record information. | 147 professionals who worked from 2019 to 2020. There were 39 professionals in the experimental group and 137 in the control group. Location: Central Catalonia, Spain - Multisite (22) | Quasi-experimental Study  Instrument:  Intervention Checklist (5) | Frequencies and percentages for categorical variables and the median with quartiles were calculated for numerical variables. Mann-Whitney test and chi-square/Fisher F test was used to compare the 2 groups' baseline information. To analyze the differences in pre-intervention and post-intervention Wilcoxon test was used. A linear regression model was also used to estimate the effect of the group on the total # of dxs. | Professionals who received the intervention labeled a higher median than those who had not, suggesting that the determinant existed but was not recorded. Comparing SDHs between the experimental group where diagnoses recorded during the 11 years prior to the intervention were also compared versus diagnoses recorded 6 months after the intervention, a significant difference (P<.001) can be observed. | Strengths include the study's focus on training providers to improve documentation of social determinants of health (SDH) in the EHR. Limitations include vague details in the study methods and a significantly smaller intervention group compared to the control group. | Providers that authored the researchers to monitor the number of SDH entries recorded over 6 months. Control group consisted of professionals who had some of the SDH labeled but did not participate in intervention. Randomization was not used. Providers came from nursing, social work, medicine, and dentistry. |
| Berger-Jenkins et al. 2019 | To implement and evaluate comprehensive screening for child behavior and social determinants of health in a pediatric primary care setting. The study aimed to explore rates of referrals and follow-up for positive screens and investigate associations between reported parental concern for their child's behavior, child behavior symptoms, and social stressors. | The screening utilized the Survey of Well Being of Young Children (SWYC), a tool was administered both on paper and electronically using tablets to enhance ease of use and confidentiality. The intervention involved routine screening using an adapted version of the SWYC for all children aged 6 months to 10 years during well-child visits. The screening tool included domains for parental concern, child behavior symptoms, and social stressors. Positive screens led to referrals and follow-up appointments with primary medical doctors (PMDs) and social workers. | 349 patient medical records was reviewed for detailed analysis of referrals and follow-ups. Location: New York, USA - Single Site | Quality improvement methodology.  Instruments:  Pediatric Symptoms Checklist 17 Patient Health Questionnaire-2 (PHQ-2) Partent's Concerns Questions The Baby Pediatric Symptom Checklist Survey of Well Being of Young Children (SWYC) | Descriptive statistics were used to analyze screen administration and documentation rates. Logistic regression was used to examine associations between parental concern and child behavior symptoms and social stressors. Data from medical records were reviewed to track referrals and follow-ups for positive screens. | The screening was initially administered using paper forms. However, to improve administration rates and address issues such as lost forms and ease of use, the study introduced an electronic method of administering the screen using tablets in the waiting room. This transition to tablet-based screening resulted in higher administration rates, reaching up to 90%. Although the tablet-based method improved administration rates, it introduced challenges in documentation. Screens administered electronically were not automatically integrated into the electronic medical record. Instead, they had to be accessed via a separate hospital clinical application, which led to lower documentation rates during this period. | Strengths include the use of technology to streamline the screening process, improve data collection, and support healthcare providers in managing psychosocial health needs in pediatric primary care. The transition to electronic screening showed potential to increase administration rates. Limitations include the need for better integration with medical record systems for effective documentation and follow-up, the single-site design limiting generalizability, lack of follow-up data for incomplete screens or those outside the convenience sample, and ongoing challenges with documentation rates. | All children aged 6 months to 10 years presenting for well-child visits were eligible for the screening. Infants younger than 6 months and adolescents over 10 years were excluded to avoid screening burden. Non-English or non-Spanish speaking families were also excluded from the study. |
| Kocielnik et al. 2019 | To design and evaluate HarborBot, a chatbot intended to improve the recording and identification of SDOH in emergency departments. The study aimed to assess the chatbot's usability and its impact on data quality and user engagement, especially among patients with low health literacy​ | HarborBot is a voice-enabled chatbot designed to facilitate social needs screening in emergency departments. It leverages natural language processing and text-to-speech technology to mimic human interaction, providing an engaging and comprehensible interface for users with varying levels of health literacy​. Participants used HarborBot to complete a social needs survey, which was also administered through a traditional survey platform (SurveyGizmo) for comparison. | 30 participants, including both high and low health literacy individuals, recruited from emergency departments and the general population in Seattle and Los Angeles. Location: Washington & California, USA - Multisite (2) | Within-subjects mixed methods study design  Instruments:  National Aeronautics and Space Administration Task Load Index (NASA TLX) Rapid Estimate of Adult Health Literacy (REALM) Newest Vital Sign (NVS) O'Brian's Engagement Survey Willingness to Share Information Social Needs Survey from Los Angeles County Health Agency (LACHA) | Quantitative analysis included calculating frequencies and percentages for categorical variables and medians with quartiles for numerical variables. Paired t-tests and linear mixed effects models were used to compare responses between HarborBot and SurveyGizmo. Qualitative data were analyzed to identify themes related to user experience and preferences​ | The study found that low health literacy participants preferred HarborBot due to its engaging and comprehensible interface, while high health literacy participants preferred the traditional survey for its efficiency. Both platforms produced comparable data quality, with an 87% equivalence in responses. HarborBot was effective in increasing engagement and comprehension among low health literacy users​ | Strengths include the potential to enhance data collection and user engagement in social needs screening, especially for low health literacy populations, through the use of a voice-enabled, conversational interface that made the tool accessible and user-friendly. Limitations include a small sample size, particularly for low health literacy participants, potential bias from non-randomized recruitment, a short study duration limiting assessment of long-term behavior changes, and feedback indicating that some participants found HarborBot to be pushy or robotic, highlighting areas for design improvement. | Participants were adults with conversational English proficiency .They were recruited from emergency departments and community centers, ensuring a diverse sample. Inclusion criteria focused on individuals with varying levels of health literacy and different demographic backgrounds |
| Langevin et al. 2023 | The purpose of this study was to evaluate the implementation fidelity of a chatbot for social needs screening in an emergency department setting. It aimed to investigate patient perceptions of the chatbot's acceptability, feasibility, and appropriateness, and assess its effectiveness in screening for social determinants of health. | The study utilized a chatbot designed to screen for social needs in the ED. The chatbot interacted with users via text and voice (output only) and was accessible on mobile phones and desktops. The chatbot used BotUI for the user interface and REDCap for data storage​. ED patients used the chatbot to complete a social needs screening questionnaire. The chatbot provided personalized recommendations for community resources based on the responses. Participants interacted with the chatbot on an iPad, with the option to use disposable headphones for privacy​ | 350 participants who ranged in age from 18 to 90 years old and were diverse in race/ethnicity, education, and insurance status. Follow-up interviews were conducted with 22 participants to gain deeper insights into their perceptions​. Location: Washington, USA - Single Site | A mixed-methods approach using concurrent triangulation.  Instruments:  CMS Accountable Health Communities Health-Related Social Needs Screening Tool Benefits Eligibility Screening Tool (BEST) Los Angeles County Health Agency (LACHA) screening guide Acceptability, Feasibility, and Appropriateness | Descriptive statistics were used to analyze survey data on demographic information and implementation measures. An inductive-deductive thematic approach was used to analyze interview transcripts, incorporating concepts from the Consolidated Framework for Implementation Research (CFIR)​ | Participants rated the chatbot as acceptable, feasible, and appropriate for social needs screening, with average ratings of 3.93 (SD=0.99) for acceptability, 4.20 (SD=0.86) for feasibility, and 4.10 (SD=0.86) for appropriateness. Participants appreciated the chatbot's responsiveness, ease of use, efficiency, and ability to provide privacy during information disclosure. Some participants had concerns about data security and desired more information on how their data would be used​. Some participants, particularly older individuals or those less familiar with technology, found the chatbot less acceptable. They preferred traditional methods of interaction and expressed discomfort with new technologies. Effective follow-up on the identified needs was deemed essential to ensure patients felt cared for and supported​ | The study's strengths include the diverse sample of participants and the real-world ED setting enhance the generalizability of the findings. The study also highlighted the chatbot's potential to engage a large, diverse patient population​. Limitations include potential self-selection bias, as those with negative experiences may have been less likely to participate, the influence of the research team’s presence and the novelty effect on participant feedback, and the single geographic region, which may limit generalizability. | at least 18 years old, spoke English or Spanish, and did not have an acute medical or psychiatric condition. Patients with an ESI score of 3-5 were included, ensuring they did not require immediate medical attention​ |
| Kormanis et al. 2024 | Determine feasibility of a sms service to screen and recommend health-related social need resources | The study employed an automated SMS campaign using cell phone numbers cataloged in the local electronic health records of hospitals at the study sites. The message included an introduction to the study and a link to a web-based screening tool. After completing the survey, participants had the option to receive resource referrals based on positively screened health-related social needs. | 200 English & Spanish adult patients with hypertension who visited one of 17 in-network clinics in Wake Forest within the last 3 months Location: North Carolina, USA - Multisite (17) | Single-arm, cross-sectional, pilot feasibility design.  CMS Accountable Health Communities Health-Related Social Needs Screening Tool | Descriptive statistics were used to analyze demographic characteristics of the sample while bivariate analyses (chi-square, fisher exact) and significance (welch’s t) tests highlighted factors that contribute to HRSN screening rates | SMS is unfeasible for screening adult patients for health-related social needs in isolation. Multiple modalities are recommended to cover a diverse demographic of patients reliably. | As a pilot, this study recruited a large sample size (n=200) for its intervention. Message content and surveys were built on feedback stakeholder groups, and the procedure is based on the RE-AIM framework. This study is limited in how the intervention engaged participants with a very low screening rate (<10%). There are no comparisons to routine clinical workflows and did not employ mitigations when phone service was not available or alternatives for SMS. Participants were also not informed about the study prior to receiving the anonymous text, leading most to distrust the message and passively abstain from study activities. | at least 18 years old, had a primary care appointment within the last 3 months at a Atrium Health Wake Forest Baptist Health clinic within Forsyth Country (17 centers), diagnosed with hypertension in the EHR, had a listed phone number in the EHR |
